# An annotated checklist of the Tortricidae of the region of Murcia (Spain) with new records, distribution and biological data (Lepidoptera, Tortricoidea)

**DOI:** 10.3897/BDJ.13.e150786

**Published:** 2025-05-23

**Authors:** Manuel J. Garre, John Girdley, Juan J Guerrero, Rosa M. Rubio, Antonio S. Ortiz

**Affiliations:** 1 University of Murcia, Murcia, Spain University of Murcia Murcia Spain

**Keywords:** Lepidoptera, Tortricidae, checklist, chorology, distribution, new records, phenology, feeding patterns, Iberian Peninsula

## Abstract

**Background:**

The Murcia Region is located at the south-east corner of the Iberian Peninsula and has a great diversity of Lepidoptera fauna, as a zoogeographical crossroads and biodiversity hotspot with almost 1,100 butterflies and moth species recorded. The study of its Microlepidoptera fauna has already been initiated previously for the families Crambidae and Pyralidae into the clade Obtectometra.

**New information:**

This document presents a detailed and critical catalogue of Tortricidae moths (Lepidoptera, Tortricoidea) from the Murcia Region, derived from an analysis of museum specimens as well as both published and new observations. A total of two subfamilies (Tortricinae and Olethreutinae), 52 genera and 107 species have been identified and are provided here, complete with their collection details, references in literature and biological information. This includes chorotype, voltinism, larval feeding behaviour and the flight periods observed within the study area. Amongst these, seventy-four species are recorded for the first time, twenty-six species are corroborated from existing literature and merely seven species have yet to be seen in the Murcia Region.

## Introduction

The Tortricidae, belonging to the superfamily Tortricoidea, are mostly nocturnal micromoths (Microlepidoptera) with an estimated 10,300 named species worldwide, of which the European fauna is represented by ca. 1,100 species (Gilligan et al. 2018) and 527 in the Iberian Peninsula (Redondo et al. 2020). The three main evolutionary lineages within Tortricidae are Totricinae, Chlidanotinae and Olethreutinae and they are monophyletically characterised by head rough-scaled above; scaling of lower frons short, appressed and upwardly directed; proboscis well developed and unscaled; labial palpi three-segmented and generally held horizontally or porrect, with apical segment short and blunt; maxillary palpi reduced; ocelli and chaetosema present; ovipositor lobes flat. The structure of the female ovipositor is the only single apomorphy that unites the entire family (Horak and Brown 1991).

The Tortricidae of the Iberian Peninsula are poorly recorded and more precise data are necessary for the production of distribution maps since only Aragon (Redondo et al. 2020), Catalonia (Ylla et al. 2011) and Portugal (Corley 2015) have their own catalogues used to update Iberian Lepidoptera catalogue from Vives-Moreno (2014) along with other more specific contributions on species (see in Redondo et al. (2020)).

Historically, the first tortricid moths recorded and described from Murcia Region in Kennel (1901) were *Euxanthisbigenerara* Kennel, 1901, currently considered synonymous with *Cochylimorphacultana* (Lederer, 1855); *Euxanthislentiginosana* Kennel, 1901, currently considered synonymous with *Cochylimorphastraminea* (Haworth, 1811); *Cochylisincommodana* Kennel, 1901, currently considered synonymous with *Aetheslanguidana* (Mann, 1855); and *Grapholitabipartitana* Kennel, 1901 and *G.dimidiatana* Kennel, 1901, both currently considered synonymous with *Grapholitalunulana* (Denis & Schiffermüller, 1775). Subsequently, Alvarez (1907) cited *Aethesperfidana* (Kennel, 1901), Caradja (1916) recorded without specifying the location of *Phtheochroarugosana* (Hübner, 1799), *Pelochristafusculana* (Zeller, 1847), *Aethesmargarotana* (Duponchel, 1836), *Xerocnephasiarigana* (Sodoffsky, 1829) and *Thiodiatrochilana* (Frölich, 1828) and Kennel (1919) described *Agapetaangelana* (Kennel, 1919) from Murcia. From the 1920s onwards, Rebel and Zerny (1927) described *Phtheochroacymatodana* (Rebel, 1927) from Sierra Espuña repeatedly re-described as *Phaloniahermosa* by Schmidt (1934) and subsequently synonymised. Schmidt (1934) also described *Paramesiaalhamana* (Schmidt, 1934) from the same locality, while Hering (1935) recorded *Selanialeplastriana* (Curtis, 1831) like *Laspeyresiavana* Kennel, 1901 from Totana and Domínguez (1943) and Ruiz-Castro (1943) mentioned several species with phytosanitary interest, such as *Aclerisvariegana* (Denis & Schiffermüller, 1775), *Cydiapomonella* (Linnaeus, 1758), *Sparganothispilleriana* (Denis & Schiffermüller, 1775) and *Lobesiabotrana* (Denis & Schiffermüller, 1775).

Later, in the second half of the 20^th^ century, Razowski (1970) recorded *Agapetaangelana* (Kennel, 1919), *Phtheochroacymatodana* (Rebel, 1927), *Aethesflagellana* (Duponchel, 1836) and *Aethestornella* (Walsingham, 1898), while *Aethesbilbaensis* (Rössler, 1877) was cited by Agenjo (1973), *Avariahyerana* (Millière, 1858) by Agenjo (1976), *Pseudococcyxtessulatana* (Staudinger, 1871) by Templado (1976) and *Grapholitamolesta* (Busck, 1916) by Garrido et al. (1979). In the 80s, Derra and Hacker (1982) recorded *Aethesmoribundana* (Staudinger, 1859), *A.margarotana* (Duponchel, 1836) and *A.flagellana* (Duponchel, 1836); Derra (1989) cited *Phtheochroasyrtana* Ragonot, 1888 and *Phtheochroaochrobasana* (Chrétien, 1915); and Baixeras and Domínguez (1993) recorded *Paramesiaalhamana* (Schmidt, 1933).

In the 21^st^ century, Razowski (2003) and Razowski (2009) referenced *Cydiagilviciliana* (Staudinger, 1859) and *Agapetaangelana* (Kennel, 1919), respectively and Larsen (2010) described *Clavigestagerti* Larsen, 2010 from Sierra Espuña amongst other Iberian localities. The last known was the invasive and polyphagous tortricid *Platynotastultana* Walsingham, 1884, economically important species native from Mexico and the south-western United States (Hymenoptera 2011, Groenen and Baixeras 2013). Particularly noteworthy is the recent description of *Eucosmacallei* Girdley, Garre, Rubio & Ortiz, 2025 from the northwest of Murcia Region (Girdley et al. 2025).

**Figure 1.**
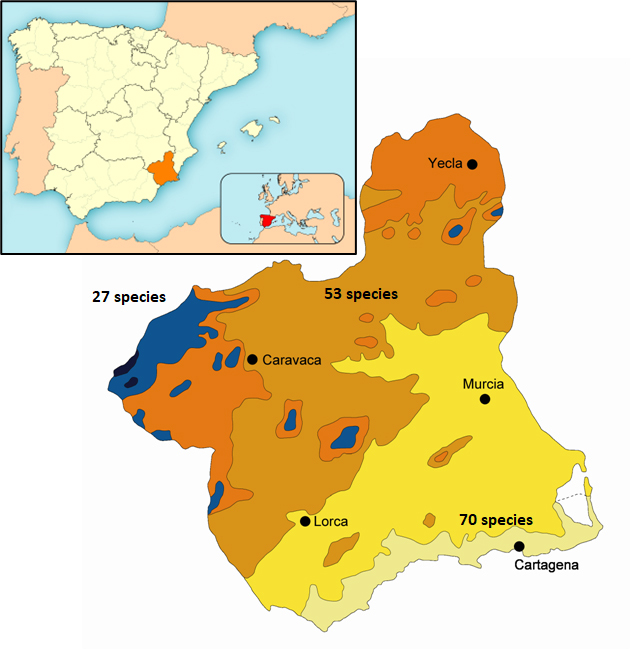
Map of the known species diversity in the bioclimatic areas in the Murcia Region. Black and blue: Oro- and Supramediteranean (27 spp.); orange and light brown: Cold and mild Mesomediterranean (53 spp.); Yelow and light green: Upper and lower Thermomediterranean (70 spp.).

The summary for the ecophysiological characterisation of the study area (south-eastern Iberian Peninsula) can be consulted in Garre et al. (2021). Considering various bioclimatic approaches relative to temperature (thermotypes) and rainfall (ombrotypes), four different bioclimatic belts can be recognised according to Alcaraz-Ariza et al. (2008): thermo-, meso-, supra- and oromediterranean (Fig. 1).

The study of the Tortricidae family of the Region of Murcia is a continuation of those initiated for the families Crambidae (Garre et al. 2021) and Pyralidae (Garre et al. 2022) and the present checklist is intended to update the recorded species and to facilitate access to the most recent data about distribution, chorology, phenology and voltinism.

## Materials and methods

**Figure 2.**
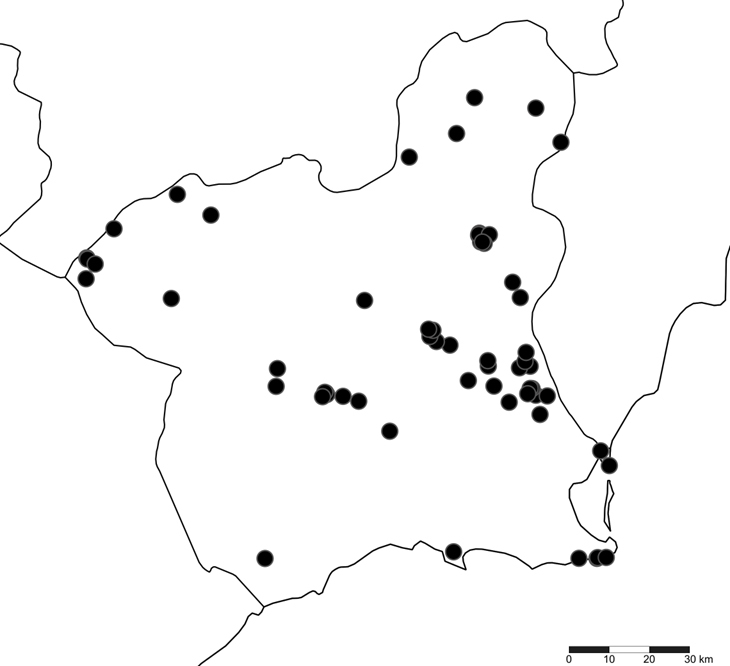
Map of the distribution of the localities sampled in the Murcia Region.

The list contains all species of Tortricidae collected by the authors along with the material deposited in the private collections of J.A. de la Calle, F. Lencina, F. Albert and F. Arcas. The study was carried out from 1978 to 2024 distributed in 424 samples taken in 57 locations belonging to 17 municipalities in the Region of Murcia (Fig. 2). It also includes all of those records previously referenced in the bibliography.

Black and actinic (6 and 15 W) Heath traps, 125 W Robinson traps, 125 W mercury vapour traps and 4 W LED light traps were used for nocturnal sampling. Catches taken in the urban environment (street lighting) are also included. All these sampling points are located within the study area and cover protected areas like the mountainous Regional Parks of Sierra Espuña and Sierra de la Pila, the coastal Regional Parks of Calblanque, Monte de las Cenizas y Peña del Águila and Salinas y Arenales de San Pedro del Pinatar, Humedal del Ajauque y Rambla Salada, Saladares del Guadalentín and Revolcadores, as well as other natural areas without special protection including agricultural landscapes and urban environments.

All studied specimens are deposited in the entomological collection in the Zoology Department of Murcia University (Spain) and in the collections of Francisco Lencina, Fernando Albert and Francisco Arcas. The occurrence data can be accessed at GBIF by https://doi.org/10.15470/lo5ekw

### Notes on the checklist

The subfamilies are systematically ordered and identified, based on the classification of Tortricidae by Gilligan et al. (2018). The genera and species are listed under their subfamilies and tribes and are also ordered systematically, together with collection data (sampling localities, altitude, decimal coordinates, date and number of specimens). In addition, for each species, related references and biological data are provided, including general chorotypes, feeding patterns of larvae, voltinism based on literature and the flight period in the study area or nearby areas indicated by months in Roman numerals.

Baixeras-Almela (1987), Razowski (2002), Razowski (2003), Razowski (2008), Razowski (2009) and Leraut (2023) were consulted to obtain the information on biology, voltinism and geographical distribution of the species, while Yela (1992) was consulted for biogeographic criteria. The Tortricidae of the Murcia Region are ordered into two subfamilies, Tortricinae and Olethreutinae and 13 tribes: Cochylini, Tortricini, Cnephasiini, Sparganothini, Archipini, Olethreutini, Eucosmini, Enarmoniini and Grapholitini (Gilligan et al. 2018).

## Checklists

### Annotated checklist of Tortricidae recorded in the Murcia Region

#### Tortricidae

DD1F3752-ADB7-53CA-B3DE-88632D8B0F8D

#### Tortricinae

A8E28354-9A0E-52C5-AAE5-0CF380261163

#### Cochylini

DD521B5D-5403-57F7-81C1-9BB6291183AA

#### Phtheochroa cymatodana

(Rebel, 1927)

5095C43F-326C-5BA4-AE37-F88FF2483127

##### Distribution

Atlanto-Mediterranean

##### Notes

References: Rebel and Zerny (1927); Schmidt (1934) as Phaloniahermosa Schmidt, 1934; Razowski (1970). Biological data: Univoltine. Flight period: V. Feeding patterns: Unknown.

#### Phtheochroa ochrobasana

(Chrétien, 1915)

F08F6192-3B25-5ABC-8F94-07373EEEFE88

##### Distribution

Atlanto-Mediterranean

##### Notes

References: Derra (1989). Biological data: Univoltine. Flight period: IX-X. Feeding patterns: Unknown.

#### Phtheochroa syrtana

(Ragonot, 1888)

7EF01C5F-1543-5521-B922-CF8F6ADC808C

##### Distribution

Mediterranean-Asiatic

##### Notes

References: Derra (1989). Biological data: Univoltine. Flight period: VIII-XI. Feeding patterns: Monophagous.

#### Phtheochroa rugosana

(Hübner, 1799)

2FBD538E-FBFE-52B6-A79D-0DAABDA565B4

##### Distribution

Mediterranean-Asiatic

##### Notes

References: Caradja (1916). Biological data: Univoltine with imagoes with a winter diapause. Flight period: II, IV-VI, IX-XI. Feeding patterns: Oligophagous.

#### Hysterophora maculosana

(Haworth, 1811)

016DC9F9-5CCE-534E-BDDD-15A0A0739216

##### Distribution

Mediterranean-Asiatic

##### Notes

Biological data: Univoltine. Flight period: IV. Feeding patterns: Oligophagous. First record in Murcia Region.

#### Cochylimorpha agenjoi

(Razowski, 1963)

FBB79141-C3DB-51A9-9D71-882C125C5454

##### Distribution

Endemic

##### Notes

Biological data: Univoltine. Flight period: X. Feeding patterns: Unknown. First record in Murcia Region.

#### Cochylimorpha cultana

(Lederer, 1855)

A800D2F3-0C5D-5F70-A608-EF42FB77A177

##### Distribution

Eurasiatic

##### Notes

References: Kennel (1901) as Euxanthisbigenerara Kennel, 1901. Biological data: Univoltine. Flight period: I-VI. Feeding patterns: Monophagous.

#### Cochylimorpha elongana

(Fischer von Röslerstamm, 1839)

69353EEE-787E-5215-95EA-CAAC2216F606

##### Distribution

Eurasiatic

##### Notes

Biological data: Bivoltine. Flight period: III-IV, IX-X. Feeding patterns: Oligophagous. First record in Murcia Region.

#### Cochylimorpha meridiana

(Staudinger, 1859)

8FDF8944-DC69-592E-B491-ECE53E51C909

##### Distribution

Eurasiatic

##### Notes

Biological data: Bivoltine. Flight period: VII-VIII. Feeding patterns: Monophagous. First record in Murcia Region.

#### Cochylimorpha decolorella

(Zeller, 1839)

67F7FC22-3174-5CF6-967D-BAE3FC038E02

##### Distribution

Mediterranean-Asiatic

##### Notes

Biological data: Univoltine. Flight period: II-III. Feeding patterns: Monophagous. First record in Murcia Region.

#### Cochylimorpha straminea

(Haworth, 1811)

0C1DB03C-F097-56E8-AADC-D078AA4E012C

##### Distribution

Eurasiatic

##### Notes

References: Kennel (1901) as Euxanthislentiginosana Kennel, 1901. Biological data: Bivoltine. Flight period: IV-VI, X-II. Feeding patterns: Monophagous.

#### Cochylimorpha salinarida

Groenen & Larsen, 2003

B56C3D84-7723-5FC9-BFAE-A5F71364799E

##### Distribution

Endemic

##### Notes

Biological data: Univoltine. Flight period: IX-X. Feeding patterns: Unknown. First record in Murcia Region.

#### Phalonidia albipalpana

(Zeller, 1847)

0FA6D726-7969-5D56-AF71-4288C8F2A796

##### Distribution

Eurasiatic

##### Notes

Biological data: Bivoltine. Flight period: III-IV, VII-IX. Feeding patterns: Monophagous. First record in Murcia Region.

#### Phalonidia contractana

(Zeller, 1847)

58ADC889-8BEC-53E6-AF12-6545FF0C220B

##### Distribution

Eurasiatic

##### Notes

Biological data: Bivoltine. Flight period: V-VI, VIII-X. Feeding patterns: Oligophagous. First record in Murcia Region.

#### Agapeta angelana

(Kennel, 1919)

F3A1FABB-A7EA-5522-8B25-286DA24C7C8D

##### Distribution

Atlanto-Mediterranean

##### Notes

References: Kennel (1919), Razowski (1970), Razowski (2009). Biological data: Bivoltine. Flight period: V-VII. Feeding patterns: Unknown.

#### Eugnosta lathoniana

(Hübner, 1800)

56349D56-457A-5E5B-8DC6-6A5B8FC98567

##### Distribution

Mediterranean-Asiatic

##### Notes

Biological data: Bivoltine. Flight period: VI, VIII-IX. Feeding patterns: Monophagous. First record in Murcia Region.

#### Aethes williana

(Brahm, 1791)

9A5D9947-AB20-55A8-8077-651C251FC787

##### Distribution

Eurasiatic

##### Notes

Biological data: Polyvoltine. Flight period: VIII-X. Feeding patterns: Polyphagous. First record in Murcia Region.

#### Aethes margarotana

(Duponchel, 1836)

06FB798A-1735-5CDB-A867-FB3285681B88

##### Distribution

Eurasiatic

##### Notes

References: Caradja (1916), Derra and Hacker (1982). Biological data: Bivoltine. Flight period: III, VI-VII. Feeding patterns: Oligophagous.

#### Aethes moribundana

(Staudinger, 1859)

81B4531E-A9EF-5750-82B5-43E4BD99A2A4

##### Distribution

Eurasiatic

##### Notes

References: Derra and Hacker (1982). Biological data: Bivoltine. Flight period: VI. Feeding patterns: Monophagous.

#### Aethes languidana

(Mann, 1855)

353A9886-79AF-5268-8702-4FF5BBCE1C87

##### Distribution

Atlanto-Mediterranean

##### Notes

References: Kennel (1901) as Cochylisincommodana Kennel, 1901. Biological data: Bivoltine. Flight period: II-IV, X. Feeding patterns: Monophagous.

#### Aethes flagellana

(Duponchel, 1836)

20EF5604-9139-5E91-9094-AB481190A07E

##### Distribution

Eurasiatic

##### Notes

References: Razowski (1970), Derra and Hacker (1982). Biological data: Univoltine. Flight period: VI. Feeding patterns: Monophagous.

#### Aethes francillana

(Fabricius, 1794)

6E7FA1C5-F8FE-5C9D-BEAC-67D1DAB2CA98

##### Distribution

Eurasiatic

##### Notes

Biological data: Bivoltine. Flight period: IV-VIII. Feeding patterns: Oligophagous. First record in Murcia Region.

#### Aethes bilbaensis

(Rössler, 1877)

4185F4FA-C7A5-54E6-A627-3686CF6534B7

##### Distribution

Eurasiatic

##### Notes

References: Agenjo (1973). Biological data: Bivoltine. Flight period: VIII, X. Feeding patterns: Oligophagous.

#### Aethes tornella

(Walsingham, 1898)

1B061BB8-1498-59B2-AE8A-E2A528C280E6

##### Distribution

Eurasiatic

##### Notes

References: Razowski (1970). Biological data: Bivoltine. Feeding patterns: Unknown.

#### Aethes scalana

(Zerny, 1927)

3C46A8EA-A4EE-5F30-B9D0-EE430A9943EE

##### Distribution

Mediterranean-Asiatic

##### Notes

Biological data: Univoltine. Flight period: VIII-IX. Feeding patterns: Unknown. First record in Murcia Region.

#### Aethes perfidana

(Kennel, 1901)

866D9D32-01F7-5093-ACA9-58633520BBF6

##### Distribution

Endemic

##### Notes

References: Alvarez (1907). Biological data: Univoltine. Feeding patterns: Unknown.

#### Aethes kindermanniana

(Treitschke, 1830)

58E08597-C4E3-5AE4-972A-4E49D9C64DC3

##### Distribution

Holarctic

##### Notes

Biological data: Bivoltine. Flight period: VIII. Feeding patterns: Oligophagous. First record in Murcia Region.

#### Cochylidia heydeniana

(Herrich-Schäffer, 1851)

7FD24552-5840-5AD4-AAA3-FE076B393F4A

##### Distribution

Eurasiatic

##### Notes

Biological data: Bivoltine. Flight period: X. Feeding patterns: Oligophagous. First record in Murcia Region.

#### Diceratura infantana

(Kennel, 1899)

7F2B3E56-1333-5A2F-9208-A68129313AD3

##### Distribution

Atlanto-Mediterranean

##### Notes

References: Razowski (1970). Biological data: Bivoltine. Flight period: VI. Feeding patterns: Unknown.

#### Longicornutia epilinana

(Duponchel, 1843)

278E2C17-159C-5F51-85C6-AFD4E4A61D3C

##### Distribution

Eurasiatic

##### Notes

References: Razowski (1970). Biological data: Bivoltine. Flight period: II, IV-VI. Feeding patterns: Monophagous.

#### Neocochylis molliculana

(Zeller, 1847)

7CA180CB-479D-5D4F-B382-09FE9AC85C40

##### Distribution

Mediterranean-Asiatic

##### Notes

Biological data: Bivoltine. Flight period: IV-VIII. Feeding patterns: Monophagous. First record in Murcia Region.

#### Pontoturania posterana

(Zeller, 1847)

133D3168-B1D4-50F4-92B5-44568614A203

##### Distribution

Eurasiatic

##### Notes

Biological data: Bivoltine. Flight period: V-VII. Feeding patterns: Oligophagous. First record in Murcia Region.

#### Tortricini

BEB1DCB0-87D1-5D19-A107-A413A4080DB9

#### Tortrix viridana

Linnaeus, 1758

F6852AB6-0FFB-5AB4-9D14-62BD5C7DF957

##### Distribution

Eurasiatic

##### Notes

Biological data: Univoltine. Flight period: VI. Feeding patterns: Monophagous. First record in Murcia Region.

#### Aleimma loeflingiana

(Linnaeus, 1758)

B61AA586-2881-59EB-8EBF-2766D376F110

##### Distribution

Eurasiatic

##### Notes

Biological data: Univoltine. Flight period: VI. Feeding patterns: Oligophagous. First record in Murcia Region.

#### Acleris variegana

(Denis & Schiffermüller, 1775)

305FBCE8-D744-5ECE-84A5-3ED2E06D48A5

##### Distribution

Holarctic

##### Notes

References: Domínguez (1943). Biological data: Univoltine with imagoes with a winter diapause. Flight period: VII-X, V. Feeding patterns: Polyphagous.

#### Cnephasiini

EF071F70-63F2-5779-A0AF-B81950D01F40

#### Xerocnephasia rigana

(Sodoffsky, 1829)

46A5E86D-92F4-553C-86F9-4CB205363246

##### Distribution

Palaearctic

##### Notes

References: Caradja (1916). Biological data: Bivoltine. Feeding patterns: Oligophagous.

#### Oxypteron schawerdai

(Rebel, 1936)

D6E5A2B8-6FFD-51C6-84D5-19CEAD934A4F

##### Distribution

Atlanto-Mediterranean

##### Notes

Biological data: Univoltine. Flight period: IX-XI. Feeding patterns: Unknown. First record in Murcia Region.

#### Eana nevadensis

(Rebel, 1929)

89991B42-B8BC-5359-B6C1-D2B442541885

##### Distribution

Atlanto-Mediterranean

##### Notes

Biological data: Univoltine. Flight period: VI-VII. Feeding patterns: Unknown. First record in Murcia Region.

#### Cnephasia pasiuana

(Hübner, 1799)

AD22D58B-84D3-5AF2-BDA6-A7F3253C4768

##### Distribution

Eurasiatic

##### Notes

Biological data: Univoltine. Flight period: V. Feeding patterns: Polyphagous. First record in Murcia Region.

#### Cnephasia alfacarana

Razowski, 1958

A7FD3712-A1FE-5FF6-A3C6-BA0E2C819379

##### Distribution

Endemic

##### Notes

Biological data: Univoltine. Flight period: V-VI. Feeding patterns: Unknown. First record in Murcia Region.

#### Cnephasia fulturata

Rebel, 1940

966FFDE5-9D5C-57B0-B103-3AEEEB8CAC87

##### Distribution

Atlanto-Mediterranean

##### Notes

Biological data: Univoltine. Flight period: V. Feeding patterns: Unknown. First record in Murcia Region.

#### Cnephasia sedana

(Constant, 1884)

050EE6E8-9A47-5E73-9EC2-707629A05BD7

##### Distribution

Eurasiatic

##### Notes

Biological data: Univoltine. Flight period: V. Feeding patterns: Polyphagous. First record in Murcia Region.

#### Sparganothini

1555E474-5574-5236-AF46-29EAC81D85F6

#### Sparganothis pilleriana

(Denis & Schiffermüller, 1775)

96A200BC-8E43-5DDA-8200-92E8B7C8E849

##### Distribution

Holarctic

##### Notes

References: Ruiz-Castro (1943). Biological data: Univoltine. Feeding patterns: Polyphagous.

#### Platynota stultana

(Walsingham, 1884)

F01F0812-7E10-52A9-9396-D36E9D87CC17

##### Distribution

Holarctic

##### Notes

References: Hymenoptera (2011). Biological data: Polyvoltine. Flight period: III-X. Feeding patterns: Polyphagous.

#### Archipini

D74F8C89-2942-57FC-A065-300C03E381A6

#### Ditula joannisiana

(Ragonot, 1889)

0D302BC8-DDAC-5FCA-8AB4-10EF578E1B87

##### Distribution

Atlanto-Mediterranean

##### Notes

Biological data: Univoltine. Flight period: X. Feeding patterns: Polyphagous. First record in Murcia Region.

#### Paramesia alhamana

(Schmidt, 1934)

61C790C2-918D-5DEB-88D7-4DEBD6DBC173

##### Distribution

Endemic

##### Notes

References: Schmidt (1934), Baixeras and Domínguez (1993). Biological data: Univoltine. Flight period: IV-V. Feeding patterns: Unknown.

#### Avaria hyerana

(Millière, 1858)

D8267680-E6B8-5ABF-A4C1-87BE41E9F2A5

##### Distribution

Mediterranean-Asiatic

##### Notes

References: Agenjo (1976) as Hastula lithosiana Kennel, 1889. Biological data: Univoltine. Flight period: X. Feeding patterns: Monophagous.

#### Pandemis heparana

(Denis & Schiffermüller, 1775)

83F96CB5-0389-51ED-A8C0-8A6F995CF285

##### Distribution

Holarctic

##### Notes

Biological data: Bivoltine. Flight period: IX. Feeding patterns: Polyphagous. First record in Murcia Region.

#### Lozotaenia cupidinana

(Staudinger, 1859)

479C1589-17D3-5B98-A571-B15E64719F64

##### Distribution

Atlanto-Mediterranean

##### Notes

Biological data: Bivoltine. Flight period: II-VII, IX-X. Feeding patterns: Polyphagous. First record in Murcia Region.

#### Cacoecimor​pha pronubana

(Hübner, 1799)

A160A21A-778D-5E3B-B248-15612413B0F7

##### Distribution

Cosmopolitan

##### Notes

Biological data: Bivoltine. Flight period: IV-VIII, X. Feeding patterns: Polyphagous. First record in Murcia Region.

#### Clepsis unicolorana

(Duponchel, 1835)

E0CEC5D0-93F1-586C-A754-C7BA794E2933

##### Distribution

Atlanto-Mediterranean

##### Notes

Biological data: Univoltine. Flight period: III-IV. Feeding patterns: Monophagous. First record in Murcia Region.

#### Clepsis eatoniana

(Ragonot, 1881)

4132D88D-E635-576A-8774-F64379EDEE9A

##### Distribution

Atlanto-Mediterranean

##### Notes

Biological data: Bivoltine. Flight period: II-VI, IX-X. Feeding patterns: Unknown. First record in Murcia Region.

#### Clepsis siciliana

(Ragonot, 1894)

4B923F42-8092-5A77-8385-782A0A46EC2D

##### Distribution

Atlanto-Mediterranean

##### Notes

Biological data: Bivoltine. Flight period: II, IV-X. Feeding patterns: Unknown. First record in Murcia Region.

#### Lozotaeniodes cupressana

(Duponchel, 1836)

F0E6720C-54CB-57D9-B61E-4EE027AE3537

##### Distribution

Mediterranean-Asiatic

##### Notes

Biological data: Bivoltine. Flight period: X. Feeding patterns: Oligophagous. First record in Murcia Region.

#### Olethreutini

C55EF005-7BE5-5231-A307-AA573F652882

#### Bactra lancealana

(Hübner, 1799)

06275920-4B1E-5809-AEE8-003327CE15F1

##### Distribution

Cosmopolitan

##### Notes

Biological data: Bivoltine. Flight period: IV-V. Feeding patterns: Polyphagous. First record in Murcia Region.

#### Bactra venosana

(Zeller, 1847)

300818CB-D150-5F3E-8510-DA0FB985AE08

##### Distribution

Cosmopolitan

##### Notes

Biological data: Bivoltine. Flight period: IV, VII-X. Feeding patterns: Monophagous. First record in Murcia Region.

#### Bactra bactrana

(Kennel, 1901)

B01907EC-EE66-5B15-8502-40408443A4D7

##### Distribution

Cosmopolitan

##### Notes

Biological data: Polyvoltine. Flight period: III-V, VII-X. Feeding patterns: Polyphagous. First record in Murcia Region.

#### Bactra simpliciana

Chrétien, 1915

472C791C-2D74-5DE8-87C6-00F712B2CC36

##### Distribution

Mediterranean-Asiatic

##### Notes

Biological data: Bivoltine. Flight period: V. Feeding patterns: Monophagous. First record in Murcia Region.

#### Endothenia oblongana

(Haworth, 1811)

3FA5E068-1D06-59C3-8D4C-1EB89933F4CA

##### Distribution

Eurasiatic

##### Notes

Biological data: Univoltine. Flight period: V-VII, X. Feeding patterns: Polyphagous. First record in Murcia Region.

#### Endothenia marginana

(Haworth, 1811)

4907B611-9EF9-52ED-B4C0-71233E27F3D0

##### Distribution

Palaearctic

##### Notes

Biological data: Bivoltine. Flight period: V-VIII. Feeding patterns; Polyphagous. First record in Murcia Region.

#### Endothenia pauperculana

(Staudinger, 1859)

6C57AFD1-667F-5D49-8806-223838135B39

##### Distribution

Atlanto-Mediterranean

##### Notes

Biological data: Bivoltine. Flight period: II, IV-V. Feeding patterns: Oligophagous. First record in Murcia Region.

#### Hedya nubiferana

(Haworth, 1811)

AC8B4279-31C2-5E98-86A4-EA5929747858

##### Distribution

Holarctic

##### Notes

Biological data: Univoltine. Flight period: VI. Feeding patterns: Polyphagous. First record in Murcia Region.

#### Piniphila bifasciana

(Haworth, 1811)

14AF5B45-FD8E-50BB-B183-CF6319AB19B4

##### Distribution

Eurasiatic

##### Notes

Biological data: Univoltine. Flight period: VI. Feeding patterns: Monophagous. First record in Murcia Region.

#### Lobesia botrana

(Denis & Schiffermüller, 1775)

A2489C19-A95D-5C76-90A4-E381217F813B

##### Distribution

Cosmopolitan

##### Notes

References: Ruiz-Castro (1943). Biological data: Polyvoltine. Flight period: II-VI, VIII. Feeding patterns: Polyphagous.

#### Lobesia indusiana

(Zeller, 1847)

EAB2076B-BD73-5DE2-8965-B569B518E050

##### Distribution

Mediterranean-Asiatic

##### Notes

Biological data: Bivoltine. Flight period: IV-VI, IX-X. Feeding patterns: Oligophagous. First record in Murcia Region.

#### Lobesia limoniana

(Millière, 1860)

57847788-2BE6-5AA9-BD0A-A5DEDC6EA0AC

##### Distribution

Atlanto-Mediterranean

##### Notes

Biological data: Bivoltine. Flight period: II-V. Feeding patterns: Monophagous. First record in Murcia Region.

#### Eucosmini

98D69D14-3158-5E0F-AF09-833BD45ACFC9

#### Thiodia trochilana

(Frölich, 1828)

0D1F945C-A9CA-50F1-B785-A374A4A81973

##### Distribution

Mediterranean-Asiatic

##### Notes

References: Caradja (1916) as Steganoptycha delitana Fisher von Röslerstamm, 1839. Biological data: Bivoltine. Flight period: III-X. Feeding patterns: Monophagous.

#### Thiodia couleruana

(Duponchel, 1835)

A87EA54E-C093-5E37-B8D1-5BC641557983

##### Distribution

Mediterranean-Asiatic

##### Notes

Biological data: Univoltine. Flight period: IV-V, VIII. Feeding patterns: Monophagous. First record in Murcia Region.

#### Acroclita subsequana

(Herrich-Schäffer, 1851)

E963C2FE-F7C2-57AB-A60C-2042E7923828

##### Distribution

Atlanto-Mediterranean

##### Notes

Biological data: Bivoltine. Flight period: II, V, X. Feeding patterns: Monophagous. First record in Murcia Region.

#### Epinotia thapsiana

(Zeller, 1847)

5DFBB562-9974-5E62-810D-07B3C447B93E

##### Distribution

Eurasiatic

##### Notes

Biological data: Bivoltine. Flight period: IV-VI, VIII. Feeding patterns: Oligophagous. First record in Murcia Region.

#### Epinotia dalmatana

(Rebel, 1891)

FA2B76FC-00E7-5845-9CFE-6E9431176CFF

##### Distribution

Mediterranean-Asiatic

##### Notes

Biological data: Univoltine. Flight period: V-VI. Feeding patterns: Monophagous. First record in Murcia Region.

#### Zeiraphera griseana

(Hübner, 1799)

68F64574-2998-5C1C-A36A-8FAD5F930069

##### Distribution

Holarctic

##### Notes

Biological data: Univoltine. Flight period: VIII. Feeding patterns: Oligophagous. First record in Murcia Region.

#### Crocidosema plebejana

Zeller, 1847

BC3920E3-574B-583E-9C8E-213D8EDBB5D7

##### Distribution

Cosmopolitan

##### Notes

Biological data: Polyvoltine. Flight period: II-X. Feeding patterns: Oligophagous. First record in Murcia Region.

#### Pelochrista fusculana

(Zeller, 1847)

743D5C45-443B-52AC-8B8B-39BD810F2770

##### Distribution

Mediterranean-Asiatic

##### Notes

References: Caradja (1916). Biological data: Univoltine. Feeding patterns: Unknown.

#### Pelochrista mollitana

(Zeller, 1847)

60C9777F-BA63-533D-8DBD-73EE2D3E0ED7

##### Distribution

Eurasiatic

##### Notes

Biological data: Univoltine. Flight period: VI. Feeding patterns: Monophagous. First record in Murcia Region.

#### Pelochrista infidana

(Hübner, 1824)

56782EE6-9987-569E-BBC0-82A1E135E5BF

##### Distribution

Eurasiatic

##### Notes

Biological data: Univoltine. Flight period: VIII-IX. Feeding patterns: Monophagous. First record in Murcia Region.

#### Eucosma cumulana

(Guenée, 1845)

6DAA2C95-7320-576A-B85D-184E0EDCF87E

##### Distribution

Mediterranean-Asiatic

##### Notes

Biological data: Univoltine. Flight period: IV. Feeding patterns: Monophagous. First record in Murcia Region.

#### Eucosma gonzalezalvarezi

Agenjo, 1970

AFCBF14F-A312-5362-B53A-319584CEF4ED

##### Distribution

Endemic

##### Notes

Biological data: Univoltine. Flight period: X. Feeding patterns: Monophagous. First record in Murcia Region.

#### Eucosma callei

Girdley, Garre, Rubio & Ortiz, 2025

5C587472-4444-55EA-82EB-AB89AC9E4744

##### Distribution

Endemic

##### Notes

References: Girdley et al. (2025). Biological data: Univoltine. Flight period: IX. Feeding patterns: Unknown.

#### Gypsonoma minutana

(Hübner, 1799)

5AD022C1-DC44-583A-99EB-0E4C4A532DCD

##### Distribution

Palaearctic

##### Notes

Biological data: Univoltine. Flight period: VII-VIII. Feeding patterns: Oligophagous. First record in Murcia Region.

#### Epiblema scutulana

(Denis & Schiffermüller, 1775)

9870FF3F-5492-5BDC-ACDA-CA5A47FD4408

##### Distribution

Palaearctic

##### Notes

Biological data: Univoltine. Flight period: V. Feeding patterns: Oligophagous. First record in Murcia Region.

#### Notocelia cynosbatella

(Linnaeus, 1758)

D08E9562-3C56-5146-95E4-D0CC4959173D

##### Distribution

Eurasiatic

##### Notes

Biological data: Univoltine. Flight period: V. Feeding patterns: Polyphagous. First record in Murcia Region.

#### Notocelia incarnatana

(Hübner, 1800)

BF9A3EE1-5C3E-5633-9D99-7E8FD6C82E9A

##### Distribution

Eurasiatic

##### Notes

Biological data: Univoltine. Flight period: IX. Feeding patterns: Monophagous. First record in Murcia Region.

#### Pseudococcyx tessulatana

(Staudinger, 1871)

54374F49-3C71-5F89-9C12-B2C0FB2AA467

##### Distribution

Mediterranean-Asiatic

##### Notes

References: Templado (1976). Biological data: Bivoltine. Flight period: II-VIII. Feeding patterns: Oligophagous.

#### Clavigesta gerti

Larsen, 2010

CAA1AA33-ADDD-5F6F-AF73-8C12CEEF0F8D

##### Distribution

Atlanto-Mediterranean

##### Notes

References: Larsen (2010). Biological data: Polyvoltine. Flight period: VII-X. Feeding patterns: Monophagous.

#### Rhyacionia buoliana

(Denis & Schiffermüller, 1775)

0CB7837E-8DC2-5DA5-A7E3-BB328B94BEA5

##### Distribution

Cosmopolitan

##### Notes

Biological data: Univoltine. Flight period: V-VIII. Feeding patterns: Monophagous. First record in Murcia Region.

#### Rhyacionia pinicolana

(Doubleday, 1850)

B141FE21-CF52-5F27-98E7-F7801D5A01E1

##### Distribution

Eurasiatic

##### Notes

Biological data: Univoltine. Flight period: VIII. Feeding patterns: Monophagous. First record in Murcia Region.

#### Rhyacionia maritimana

Pröse, 1981

3C828892-FF90-5689-A2D8-FC1EB3D6C2C3

##### Distribution

Atlanto-Mediterranean

##### Notes

Biological data: Univoltine. Flight period: IV-VI. Feeding patterns: Unknown. First record in Murcia Region.

#### Rhyacionia pinivorana

(Lienig & Zeller, 1846)

EB399549-4B20-59EA-A2FB-302144AA47A6

##### Distribution

Eurasiatic

##### Notes

Biological data: Univoltine. Flight period: VI. Feeding patterns: Monophagous. First record in Murcia Region.

#### Enarmoniini

5439C3D6-23DD-53A2-B65C-462BA5A2F1B8

#### Ancylis sparulana

(Staudinger , 1859)

FB26AD40-6273-5BFB-B864-2B3ADEB8D0EC

##### Distribution

Atlanto-Mediterranean

##### Notes

Biological data: Polyvoltine. Flight period: II-VIII, X. Feeding patterns: Monophagous. First record in Murcia Region.

#### Grapholitini

C511DDDD-CD30-55CF-A4F3-5922B90DA2DF

#### Grapholita lunulana

(Denis & Schiffermüller, 1775)

65057A54-0EE8-56B1-8F3E-69F3D94D0F39

##### Distribution

Eurasiatic

##### Notes

References: Kennel (1901) as G.bipartitana Kennel, 1901 and G.dimidiatana Kennel, 1901. Biological data: Univoltine. Feeding patterns: Oligophagous.

#### Grapholita molesta

(Busck in Quaintance & Wood, 1916)

33A9A653-5714-5239-956B-A496B22CFCE2

##### Distribution

Cosmopolitan

##### Notes

References: Garrido et al. (1979). Biological data: Polyvoltine. Flight period: VI, IX. Feeding patterns: Polyphagous.

#### Cydia gilviciliana

(Staudinger, 1859)

75C66043-39AB-53D1-BCF2-77E8A27D3086

##### Distribution

Atlanto-Mediterranean

##### Notes

References: Razowski (2003). Biological data: Univoltine. Feeding patterns: Unknown.

#### Cydia ulicetana

(Haworth, 1811)

E57E88A4-F4E5-5D15-90D7-277A95967B9A

##### Distribution

Mediterranean-Asiatic

##### Notes

Biological data: Bivoltine. Flight period: IV-VI. Feeding patterns: Oligophagous. First record in Murcia Region.

#### Cydia ilipulana

(Walsingham, 1903)

8073BF22-EA69-5D38-BE78-D1A338530D8A

##### Distribution

Eurasiatic

##### Notes

Biological data: Bivoltine. Flight period: IV-VI, IX-X. Feeding patterns: Monophagous. First record in Murcia Region.

#### Cydia vallesiaca

(Sauter, 1968)

26C37097-C52E-5610-86A0-63783385DA63

##### Distribution

Atlanto-Mediterranean

##### Notes

Biological data: Univoltine. Flight period: III-VII. Feeding patterns: Monophagous. First record in Murcia Region.

#### Cydia adenocarpi

(Ragonot, 1875)

CEF00036-7B76-536C-A18A-1AC24E80F351

##### Distribution

Mediterranean-Asiatic

##### Notes

Biological data: Bivoltine. Flight period: V-VI. Feeding patterns: Monophagous. First record in Murcia Region.

#### Cydia conicolana

(Heylaerts, 1874)

1CEB8051-D5F6-5D58-9381-A929BDB43279

##### Distribution

Mediterranean-Asiatic

##### Notes

Biological data: Univoltine. Flight period: VI. Feeding patterns: Monophagous. First record in Murcia Region.

#### Cydia coniferana

(Saxesen, 1840)

EADC5689-3449-5BBE-8B5E-D3EEEA2E72F2

##### Distribution

Holarctic

##### Notes

Biological data: Bivoltine. Flight period: VI, IX. Feeding patterns: Oligophagous. First record in Murcia Region.

#### Cydia pomonella

(Linnaeus, 1758)

CB4AD1E8-4C13-5C2C-8BF9-38C131D57DE4

##### Distribution

Cosmopolitan

##### Notes

References: Domínguez (1943). Biological data: Bivoltine. Flight period: V-IX. Feeding patterns: Oligophagous.

#### Cydia splendana

(Hübner, 1799)

10303278-F9E4-519D-A9DE-5D939A6D4DC7

##### Distribution

Eurasiatic

##### Notes

Biological data: Univoltine. Flight period: X. Feeding patterns: Oligophagous. First record in Murcia Region.

#### Cydia fagiglandana

(Zeller, 1841)

B060620B-7769-5BCC-A9D9-1C79F7984E98

##### Distribution

Eurasiatic

##### Notes

Biological data: Bivoltine. Flight period: VI-X. Feeding patterns: Oligophagous. First record in Murcia Region.

#### Cydia amplana

(Hübner, 1799)

5BA5FEFB-D11D-5F1D-A867-67415D8F968F

##### Distribution

Eurasiatic

##### Notes

Biological data: Univoltine. Flight period: VIII-X. Feeding patterns: Polyphagous. First record in Murcia Region.

#### Selania leplastriana

(Curtis, 1831)

D987764D-9344-5EE8-A592-42809CF46B1A

##### Distribution

Eurasiatic

##### Notes

References: Hering (1935) as Laspeyresiavana Kennel, 1901. Biological data: Polyvoltine. Flight period: III-VIII, X-XI. Feeding patterns: Oligophagous.

#### Selania resedana

(Obraztsov, 1959)

D17519C1-1279-550F-81A5-65CBDEABC1B2

##### Distribution

Atlanto-Mediterranean

##### Notes

Biological data: Bivoltine. Flight period: V-VI. Feeding patterns: Monophagous. First record in Murcia Region.

#### Selania capparidana

(Zeller, 1847)

72EAB0F9-447F-5540-B73C-DD651B03A656

##### Distribution

Mediterranean-Asiatic

##### Notes

Biological data: Polyvoltine. Flight period: V. Feeding patterns: Monophagous. First record in Murcia Region.

#### Pammene fasciana

(Linnaeus, 1761)

FC5CCC12-2F49-531E-A4A8-0DFC19B672B8

##### Distribution

Eurasiatic

##### Notes

Biological data: Univoltine. Flight period: VI. Feeding patterns: Oligophagous. First record in Murcia Region.

## Analysis

The list includes 107 species in 52 genera and two subfamilies: Tortricinae (54 species) and Olethreutinae (53 species). Seventy-four new records (69.2%) from the Murcia Region are added to its Lepidopteran fauna. The number of species known from this territory, at this time, accounts for 9.7% of the European (1,100) and 20.3% of the Iberian species (527).

The most species-rich subfamily, Tortricinae, comprises 55.8% of all genera and 50.5% of all species, while Olethreutinae comprises 44.2% and 49.5%, respectively (Table 1).

Known Tortricidae diversity in the Murcia Region seems still relatively poor when compared to those in other Iberian Regions and with the whole of the Iberian Peninsula, as, for instance, the only three Iberian regions extensively surveyed like Catalonia (346 species; Ylla et al. (2019)), Aragon (320 species; Redondo et al. (2020)) and the whole of Portugal (235 species; Corley (2015)). However, increasing the sampling effort will allow adding new species to the checklist of the Tortricidae of the Murcia Region.

The most species-rich Tortricidae genera in the Murcia Region are *Aethes* and *Cydia* (11 species, 10.3% each, respectively), *Cochylimorpha* (7 species, 6.5%) and *Phtheochroa*, *Cnephasia*, *Bactra* and *Rhyacionia* (4 species, 3.7% each, respectively). On the other hand, eleven of the studied genera are species-poor (2-3 species) and 34 genera are single species.

Species richness varies substantially amongst the different bioclimatic belts of the Murcia Region (Fig. 1). The Thermomediterranean belt has the most diverse Tortricidae fauna with 70 species recorded, followed by the Mesomediterranean belt with 53 species, while the Supra- and Oromediteranean areas appear to be less diverse with 27 species (Table 2). In each of these belts, 34 species are unique in the Thermo-, 16 in Meso- and nine in Supra and Oromediterranean belts, while 32 species were recorded in two belts and nine in all studied belts. Thus, according with these data, excluding the seven species known only from bibliographic references (*Aethestornella*, *A.perfidana*, *Xerocnephasiarigana*, *Sparganothispilleriana*, *Pelochristafusculana*, *Cydiagilviciliana* and *Grapholitalunulana*), 55.1% of the species can be considered specialists in a given bioclimatic belt, while the other 44.9% can be considered as opportunists of different types of vegetation that characterise each of the bioclimatic belts. The detailed data for the bioclimatic belts of Tortricidae in the Murcia Region are summarised in Table 2.

Chorological analysis for the family Tortricidae in the Murcia Region showed that the species of wide distribution chorotypes are most abundant at 53.3% of the total, including the Eurasiatic (33.6%), Cosmopolitan (8.4%), Holarctic (7.5%) and Palaearctic (3.7%) chorotypes. On the other hand, the Mediterranean species are also abundant with 46.7%, distributed amongst the Atlanto-Mediterranean (20.6%), Asiatic-Mediterranean (19.6%) and endemism (6.5%) chorotypes. These last results differ from those obtained in the study of the Crambidae and Pyralidae families where the Mediterranean species were the most abundant with 56.6% (Garre et al. 2021) and 59.2% (Garre et al. 2022), respectively, which was consistent with the geographical position of the study area, while the species with wide distribution (53.3%) are the most common in the mountainous biotopes of the centre and north of the study area.

Regarding the biology of the species, the environmental conditions of the study area, which affect the availability of trophic resources for reproduction, suggest that most of the species are univoltins (49.5%) and bivoltins (41.1%), while a small proportion are polyvoltins (9.3%). The voltinism of some species is based on known data from central and northern European species, so the life cycles of species in warmer and drier regions in the south of the continent, as is the case in the Murcia Region, do not necessarily have the same characteristics. In this way, the flight period of many species may be earlier, more extended and even bi- and polyvoltinism may be more frequent due to the bioclimatic conditions of the Murcia Region.

In relation to the feeding strategies of caterpillars, 37.4% of the species are monophagous, 26.2% oligophagous, 17.8% polyphagous and the feeding behaviour for the remaining 18.7% is unknown. Approximately 20% of the listed species are opportunistic with great agricultural and forestry interest as they are considered pests of numerous cultivated and wild plants that dominate part of the Murcian territory. Amongst the species that feed on cultivated plants are *Lobesiabotrana* and *Sparganothispilleriana* feeding on vines; *Cnephasiapasiuana* on wheat; *Cacoecimorphapronubana* on pomegranate and ornamental plants; *Crocidosemaplebejana* on corn and cotton plants; *Grapholitamolesta*, *Cydiapomonella* and *Pandemisheparana* on fruit trees; *Cydiasplendana*, *C.fagiglandana* and *Pammenefasciana* on walnut trees; *Selanialeplastriana* on cabbage. Species feeding on forest and wild crops are *Rhyacionabuoliana* and *Tortrixviridana* which can cause damage to pine and oak forests, respectively. *Platynotastultana*, originally from Mexico and the United States, is the case of a recently introduced pest. Detected in Europe for the first time in Murcia in 2009 by the Plant Health Services of Murcia, it was later found in crops in Almeria, Alicante and Granada. It is a polyphagous species that feeds on a wide variety of plant species, such as vines, pomegranates, ornamental plants, stone and seed fruit trees, alfalfa, lettuce, peppers, tomatoes, etc. (Hymenoptera 2011, Groenen and Baixeras 2013), being observed in our samplings with extraordinary abundance in lemon tree crops.

On the other hand, some species are expanding their distribution in the Iberian Peninsula, such as *Cochylimorphasalinarida*, described with specimens collected in the Province of Alicante (Groenen and Larsen 2003) and extending its distribution area to some inland salt marshes; *Bactrasimpliciana*, referenced from the coast of Almeria (Gastón et al. 2018, Garre et al. 2022) and recorded in the coastal dune systems; the Iberian endemic *Eucosmagonzalezalvarezi*, known from the Provinces of Almeria, Granada, Huesca, Lerida, Madrid and Zaragoza (Šumpich 2011, Ylla et al. 2015, Cifuentes 2019); and *Selaniacapparidana*, recorded for the first time in Almeria (Clarke 2011), has been collected in an inland steppe habitat. Finally, the description of a new species, *Eucosmacallei* Girdley, Garre, Rubio & Ortiz, 2025, known so far only in the Murcia Region.

## Discussion

Prior to our investigation, the number of known Tortricidae moth species in the Murcia Region was 32. Our study increases this number to a total of 107, based on an examination of museum specimens, published records and sampled individuals, accounting for 20.3% of all of the Iberian species known. This study presents an updated checklist of current Tortricidae moth species with their distribution and biological information for the Murcia Region in the south-eastern Iberian Peninsula. This study serves as both a guide for collection in the poorly-sampled south-western European continent and a comprehensive reference list with the Tortricidae taxa and localities where conservation is an important priority for policy-makers, conservation planners and for the management of insect diversity in Spain. We encourage lepidopterists holding additional data on systematically collected tortricids to produce an updated dataset.

## Figures and Tables

**Table 1.** Numbers and percentages of known genera and species recorded for each subfamily in Murcia Region.

**Subfamilies**	**Genus richness**	% **Genus**	**Species richness**	% **Species**
Tortricinae	29	55.8	54	50.5
Olethreutinae	23	44.2	53	49.5
**Total**	52	100	107	100

**Table 2.** List of unique species in each bioclimatic area or in more than one bioclimatic area.

Oro- and Supramediterranean	*Aethesmoribundana* (Staudinger, 1859)
	*Aethesflagellana* (Duponchel, 1836)
	*Pontoturaniaposterana* (Zeller, 1847)
	*Eananevadensis* (Rebel, 1929)
	*Piniphilabifasciana* (Haworth, 1811)
	*Epiblemascutulana* (Denis & Schiffermüller, 1775)
	*Rhyacioniapinicolana* (Doubleday, 1850)
	*Rhyacioniapinivorana* (Lienig & Zeller, 1846)
	*Cydiaconicolana* (Heylaerts, 1874)
Mesomediterranean	*Phtheochroacymatodana* (Rebel, 1927)
	*Agapetaangelana* (Kennel, 1919)
	*Aethesscalana* (Zerny, 1927)
	*Aetheskindermanniana* (Treitschke, 1830)
	*Diceraturainfantana* (Kennel, 1899)
	*Aleimmaloeflingiana* (Linnaeus, 1758)
	*Cnephasiapasiuana* (Hübner, 1799)
	*Cnephasiafulturata* Rebel, 1940
	*Ditulajoannisiana* (Ragonot, 1889)
	*Paramesiaalhamana* (Schmidt, 1934)
	*Pandemisheparana* (Denis & Schiffermüller, 1775)
	*Hedyanubiferana* (Haworth, 1811)
	*Eucosmacallei* Girdley, Garre, Rubio & Ortiz, 2025
	*Notoceliacynosbatella* (Linnaeus, 1758)
	*Notoceliaincarnatana* (Hübner, 1800)
	*Cydiasplendana* (Hübner, 1799)
Thermomediterranean	*Phtheochroarugosana* (Hübner, 1799)
	*Hysterophoramaculosana* (Haworth, 1811)
	*Cochylimorphaagenjoi* (Razowski, 1963)
	*Cochylimorphaelongana* (Fischer von Röslerstamm, 1839)
	*Cochylimorphasalinarida* Groenen & Larsen, 2003
	*Phalonidiaalbipalpana* (Zeller, 1847)
	*Aetheswilliana* (Brahm, 1791)
	*Aetheslanguidana* (Mann, 1855)
	*Aethesbilbaensis* (Rössler, 1877)
	*Cochylidiaheydeniana* (Herrich-Schäffer, 1851)
	*Neocochylismolliculana* (Zeller, 1847)
	*Oxypteronschawerdai* (Rebel, 1936)
	*Cnephasiasedana* (Constant, 1884)
	*Platynotastultana* (Walsingham, 1884)
	*Avariahyerana* (Millière, 1858)
	*Lozotaeniodescupressana* (Duponchel, 1836)
	*Bactravenosana* (Zeller, 1847)
	*Bactrabactrana* (Kennel, 1901)
	*Bactrasimpliciana* Chrétien, 1915
	*Endotheniaoblongana* (Haworth, 1811)
	*Endotheniamarginana* (Haworth, 1811)
	*Endotheniapauperculana* (Staudinger, 1859)
	*Lobesiabotrana* (Denis & Schiffermüller, 1775)
	*Lobesiaindusiana* (Zeller, 1847)
	*Lobesialimoniana* (Millière, 1860)
	*Thiodiacouleruana* (Duponchel, 1835)
	*Acroclitasubsequana* (Herrich-Schäffer, 1851)
	*Zeirapheragriseana* (Hübner, 1799)
	*Eucosmacumulana* (Guenée, 1845)
	*Gypsonomaminutana* (Hübner, 1799)
	*Grapholitamolesta* (Busck in Quaintance & Wood, 1916)
	*Cydiaadenocarpi* (Ragonot, 1875)
	*Selaniaresedana* (Obraztsov, 1959)
	*Selaniacapparidana* (Zeller, 1847)
Oro-, Supra- and Mesomediterranean	*Cochylimorphameridiana* (Staudinger, 1859)
	*Eugnostalathoniana* (Hübner, 1800)
	*Cnephasiaalfacarana* Razowski, 1958
	*Cydiaulicetana* (Haworth, 1811)
	*Pammenefasciana* (Linnaeus, 1761)
Meso- and Thermomediterranean	*Phtheochroasyrtana* Ragonot, 1888
	*Cochylimorphadecolorella* (Zeller, 1839)
	*Cochylimorphastraminea* (Haworth, 1811)
	*Aethesmargarotana* (Duponchel, 1836)
	*Aethesfrancillana* (Fabricius, 1794)
	*Aclerisvariegana* (Denis & Schiffermüller, 1775)
	*Clepsiseatoniana* (Ragonot, 1881)
	*Bactralancealana* (Hübner, 1799)
	*Thiodiatrochilana* (Frölich, 1828)
	*Epinotiathapsiana* (Zeller, 1847)
	*Epinotiadalmatana* (Rebel, 1891)
	*Crocidosemaplebejana* (Zeller, 1847)
	*Pelochristainfidana* (Hübner, 1824)
	*Eucosmagonzalezalvarezi* Agenjo, 1970
	*Pseudococcyxtessulatana* (Staudinger, 1871)
	*Clavigestagerti* Larsen, 2010
	*Rhyacioniabuoliana* (Denis & Schiffermüller, 1775)
	*Ancylissparulana* (Staudinger, 1859)
	*Cydiailipulana* (Walsingham, 1903)
	*Cydiavallesiaca* (Sauter, 1968)
	*Cydiapomonella* (Linnaeus, 1758)
	*Cydiaamplana* (Hübner, 1799)
	*Selanialeplastriana* (Curtis, 1831)
Oro- and Supra- and Thermomediterranean	*Phtheochroaochrobasana* (Chrétien, 1915)
	*Tortrixviridana* Linnaeus, 1758
	*Pelochristamollitana* (Zeller, 1847)
	*Cydiaconiferana* (Saxesen, 1840)
All areas	*Cochylimorphacultana* (Lederer, 1855)
	*Phalonidiacontractana* (Zeller, 1847)
	*Longicornutiaepilinana* (Duponchel, 1843)
	*Lozotaeniacupidinana* (Staudinger, 1859)
	*Cacoecimorphapronubana* (Hübner, 1799)
	*Clepsisunicolorana* (Duponchel, 1835)
	*Clepsissiciliana* (Ragonot, 1894)
	*Rhyacioniamaritimana* Pröse, 1981
	*Cydiafagiglandana* (Zeller, 1841)
